# The current landscape and future of tablet-based cognitive assessments for children in low-resourced settings

**DOI:** 10.1371/journal.pdig.0000196

**Published:** 2023-02-23

**Authors:** Megan S. McHenry, Debarati Mukherjee, Supriya Bhavnani, Amir Kirolos, Joe D. Piper, Maria M. Crespo-Llado, Melissa J. Gladstone

**Affiliations:** 1 Department of Pediatrics, Indiana University School of Medicine, Indianapolis, United States of America; 2 Indian Institute of Public Health—Bengaluru, Life Course Epidemiology, Bengaluru, Karnataka, India; 3 Child Development Group, Sangath, India; 4 Department of Women and Children’s Health, Institute of Life Course and Medical Sciences, University of Liverpool, Liverpool, United Kingdom; 5 Blizard Institute, Queen Mary University of London, London, United Kingdom; Research Institute of the McGill University Health Centre, McGill University, CANADA

## Abstract

Interest in measuring cognition in children in low-resourced settings has increased in recent years, but options for cognitive assessments are limited. Researchers are faced with challenges when using existing assessments in these settings, such as trained workforce shortages, less relevant testing stimuli, limitations of proprietary assessments, and inadequate parental knowledge of cognitive milestones. Tablet-based direct child assessments are emerging as a practical solution to these challenges, but evidence of their validity and utility in cross-cultural settings is limited. In this overview, we introduce key concepts of this field while exploring the current landscape of tablet-based assessments for low-resourced settings. We also make recommendations for future directions of this relatively novel field. We conclude that tablet-based assessments are an emerging and promising method of assessing cognition in young children. Further awareness and dissemination of validated tablet-based assessments may increase capacity for child development research and clinical practice in low-resourced settings.

## Author summary

Tools that measure cognitive skills are key to identifying children in need of critical interventions to reach their full potential. However, there are barriers inherent in the application of traditional tools, such as the need for trained professionals, the relevance of the testing items, and the time needed to complete the evaluations. This disproportionately impacts populations living in low-resourced settings. Emerging research indicates that tablet computers can easily administer essential cognitive testing across low-resourced settings and overcome many of the barriers from the use of traditional tools. However, no resources are available that succinctly review the key considerations and available options for tablet-based cognitive assessments. Therefore, in this review, we summarize the existing known tablet-based cognitive assessments used in low-resourced settings and evaluate a myriad of factors important for the application of computerized cognitive assessments across different cultural contexts. We also offer recommendations for the future output of tablet-based assessments, which may mediate the gap for use of cognitive testing within low-resourced settings. Overall, we determine that tablet-based assessments are a promising solution among these settings, and further insight and awareness of these tools may increase their utility.

## 1. Introduction

As global focus shifts from ensuring children survive to enabling them to thrive, millions of children living in low-resourced settings are found to be at-risk of not attaining their full developmental potential [1]. This is due to their disproportionately high exposure to risk factors for poor development during critical periods in childhood [2], including malnutrition, iron-deficiency anemia, infectious diseases, exposure to violence, exposure to toxins, extreme poverty, maternal depression, and inadequate cognitive stimulation [3]. While interventions and programs are being implemented to address these risk factors [4,5], we need to understand how best to optimize neurocognitive outcomes within the context of scarce resources.

Healthy child development comprises a complex interplay of rapid physiological, psychological, and physical changes in response to early environmental experiences, with lasting effects on multiple domains of development, including cognition. Cognitive development in preschool years, through which children develop the skills to acquire, assimilate and apply knowledge, has been demonstrated to be predictive of later outcomes such as IQ and academic achievement [6,7]. Executive function, a key component of cognitive development, is our ability to temporarily manipulate information mentally (working memory), generate different solutions to a problem (cognitive flexibility), and maintain impulse control (inhibition). Executive function enables us to plan, focus attention, set and achieve goals [8], and it predicts math, reading, and science achievements in school [9–11]. Deficits in cognitive ability and executive function have long-term impacts across the life-course, starting with poor educational attainment and resulting in loss of adult income, thus impacting the livelihood of families, communities, and countries [4]. Therefore, high-quality measures of cognitive development, including executive functioning, are essential to identify children at-risk of not attaining their full developmental potential, triaging them into timely interventions, and finally, monitoring their impact on developmental trajectories within clinical, research, or community-based settings.

Many tools have been used in measurement of cognition and in some cases, executive function, in low-resource settings. Assessing cognitive ability, and in particular, executive function, in children within these settings, may be impeded by a number of challenges. These challenges include using tools with appropriate cultural and language adaptations, training a workforce for high-quality administration, and utilizing tools with adequate reliability and validity, while maintaining affordability and scalability [12,13].

Currently, many neurocognitive assessments depend on observations of child behavior conducted by child development and neuropsychology specialists, who undergo substantial training to become proficient in high quality and consistent administration, scoring and interpretation of assessment tools. Although capacity building and infrastructure in psychological testing has improved in recent years, many countries still have few trained workers qualified to implement these tools [14]. Examples of tools used for measuring cognition are the Kaufman-ABC or the Wechsler Preschool and Primary Scale of Intelligence (WPPSI) [15,16] and in younger children, the Bayley Scales for Infant and Toddler Development [17]. Measurement of executive function has been measured with tools including, but not limited to, the NEuroPSYchological Asssessment (NEPSY) [18,19], the knock-tap tests [20], the Spin the Pots task [21], or parent report measures such as the Behavior Rating Inventory of Executive Function (BRIEF) [22]. These all vary in reliability and validity [13]. Most of these tools have been developed and validated within high-resource settings, although some attempts have been made to develop versions for other settings. This can present further challenges, as most items in neuropsychological assessment tools are contextually related to settings where tools were developed and validated, therefore requiring lengthy and expensive adaptation process for use cross-culturally. This has been attempted in some settings, e.g., Kilifi, Kenya, where a battery was made for an African context; however, difficulties remain in capacity to scale-up training, application, and measurement [23]. Furthermore, tools created in high-resource contexts are often proprietary and the cost to administer in low-resourced settings can be prohibitive. There is, thus, a paucity of contextually relevant scalable cognitive assessment tools developed, validated, and used within low-resourced settings [24]. We define low-resourced settings as those with a population facing health inequities that negatively impact child development, typically in low- and middle-income countries.

Advances in technology, including the emergence of tablet computers, could be leveraged to aid in the scalable evaluation of cognition and particularly executive function, across a wide range of settings. Tablet computers are readily available, inexpensive, portable, and have functionality without internet. Paper-based childhood developmental assessment tools are increasingly being deployed on tablet computers using basic open data kits or similar programs. This includes the Malawi Developmental Assessment Tool (MDAT) [25] and the Global Scales of Early Development (GSED) [26]. While this standardizes administration and scoring by minimizing errors, these tools either rely on parent report, assuming parental knowledge of developmental milestones, or behavioral observations of children by non-specialists, which itself is resource-intensive and only partially addresses the workforce challenge.

In recent years, computerized tasks within tablet computers are increasingly being used to evaluate child performance directly. This has the potential to overcome many current challenges of measuring cognition in children globally. These tools permit the administration and scoring of a broad array of tasks that measure specific domains of cognitive functioning with minimal potential for error, while also having the potential to be gamified [27], increasing children’s interest in these cognitive tasks. Furthermore, with cameras (to capture images and videos during task performance), accelerometers and gyroscopes (to estimate motion and force on the screen) [19,20], timers (to assess latency in responses) [28], and microphones (to capture audio), developers can tap into a wide range of child responses to assess their cognitive abilities [29]. Such nuanced variables are not feasibly evaluated with traditional pen-and-paper administration of cognitive assessment tools [30,31]. Because the cognitive tasks are administered with a tablet computer rather than skilled observation of child behavior, it requires very little workforce support for administration or scoring, enabling non-specialists with minimal training to administer tasks. While smartphone technology has similar strengths, the smaller screen size presents some limitations and, at this point, only a few cognitive tools are under development for smartphone use. In sum, tablet computers provide opportunities to perform high-quality cognitive assessments of children in a manner that is easily scalable and practical in low-resourced settings.

While this technology appears to be the future of cognitive testing, no known summary of these tools exists. This overview **aims to summarize the current landscape of tablet-based cognitive assessment tools** used in low-resourced settings that directly measure child performance. We pose **considerations for use of these tools with the existing state of the evidence and make recommendations** for ways in which this novel field can move forward. This overview is for those who may want to use tools for programmatic and research evaluation and therefore specifically targets information on the feasibility and validity of the tools in their current format, when used in low-resource settings.

## 2. Methodology for compiling tools

We identified tablet-based cognitive assessment tools by performing a general scoping literature search as well as through reaching out to international content experts working in the field. We aimed to identify peer-reviewed articles providing information on tablet-based cognitive assessment tools from electronic databases that included PubMed and Google Scholar identifying any publications from January 2000 to September 2021. The search terms used included “cognition”, “executive function,” “pediatrics,” “tabled-based assessments,” and “children.” Inclusion criteria required that the assessment had to measure cognition was used in children under 18 years of age, was used in low-resourced settings, and was used on a tablet computer. Tools were excluded if they were only used in well-resourced settings or performed only on smartphones or laptop/desktop computers. Much of the information gathered required direct interviews with the developers and was not available through peer-reviewed articles. We therefore supplemented our literature search through general search engines, such as Google and Bing, and sought topic experts (i.e., those who lead and consult on projects measuring cognition in low-resourced settings, but do not themselves develop tools) to identify further tools. We emailed each application developer requesting an interview and further information about any other known tablet-based cognitive assessment tools used in low-resourced settings. Through our review of published papers, websites, and interviews with developers, we collected data on domains of cognition and executive function measured, time for training and administration, country of use and present adaptations, psychometric properties of each tool (as published to date), and feasibility of use.

## 3. Current landscape of tablet-based cognitive assessment tools for children

**Fig 1:**
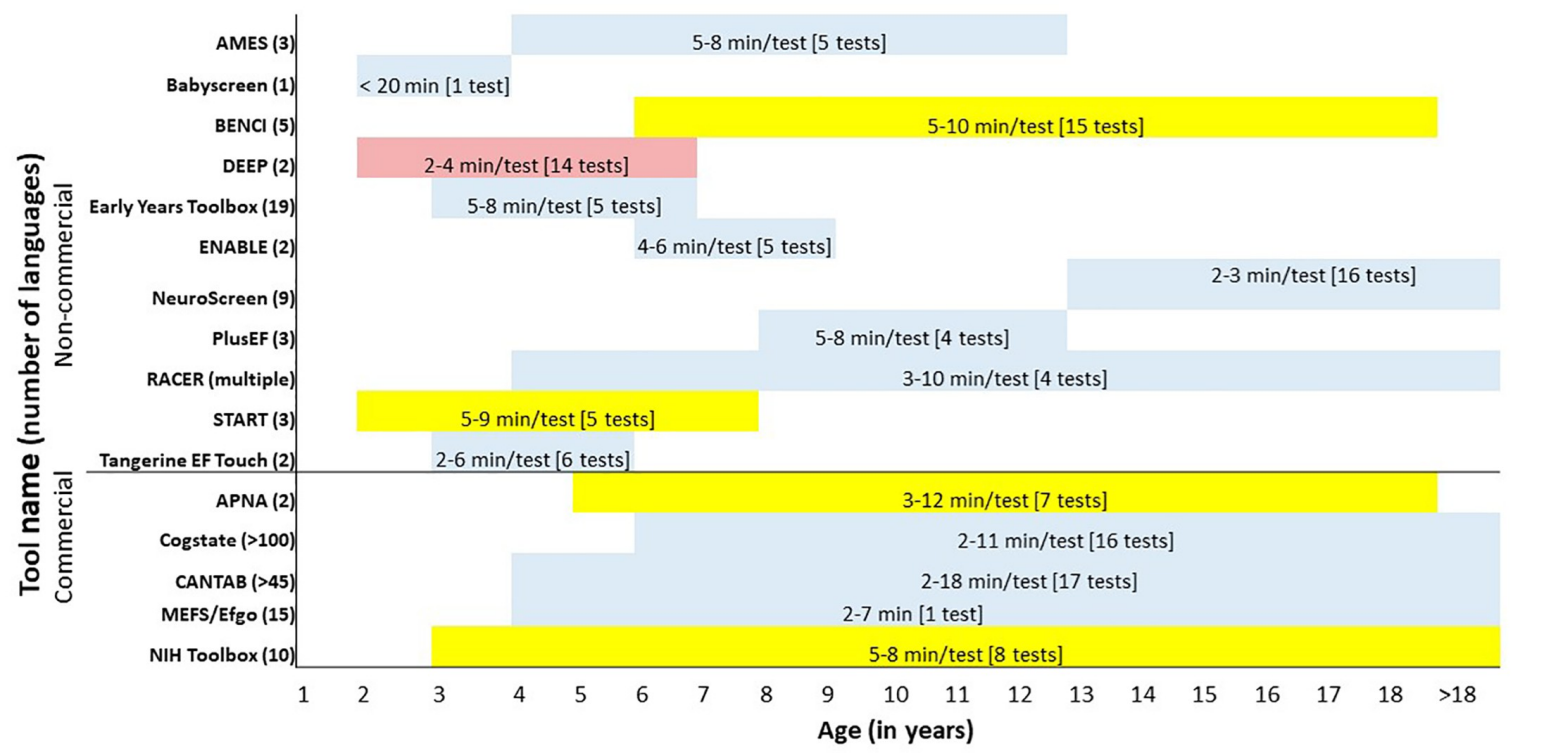
Synthesis across tablet-based cognitive assessments. The names of the tools are listed in the Y-axis, with non-commercial tools listed first, followed by commercial tools. The X-axis is the range of ages, in years, for the intended population for the tool’s use. The numbers encompassed by () by the tool names indicates the number of languages for which the tool is translated. The numbers within the bands indicate the number of minutes (min) required to complete each test, with the number of tests listed in []. The color of the bands indicates the amount of training required to administer, with blue indicating <4 h of required training, yellow indicating an estimated 4–12 h of required training, pink indicating an estimated >12 h of required training.

Numerous commercial and non-commercial tablet-based cognitive assessment tools have been developed in recent years. From our search, we identified 16 tools and described characteristics of each in Table 1 and Fig 1. Most tools were developed in North America, Europe, or Australia and initially used the English language, which was subsequently translated into other languages. Two tools had significant development work performed in India (DEEP and START), which included formative work in the community and iterative development of the user-interface through testing with non-specialist administrators and the target age range of children [32]. One tool has been further developed in Brazil (Educational Neuroscience App-Based Learning Environment (ENABLE). From interviews with the tool developers, the primary users of these tools, to date, are researchers, with administration being performed by research staff or community health workers. However, some tools, such as the Early Years Toolbox and NIH Toolbox, have also been used by educators and clinicians as well.

**Table 1:** Characteristics of tablet-based cognitive assessments.

Tool name	Year created	Countries used	Languages	Age ranges	Published validity testing*	Approximate time to administer	Training require-ments †
Non-commercial tools							
AMES	2018	United States, Ghana, Ivory Coast	English, Ivoirian French	4–12 years	No	Variable; up to 5 tasks can be used, and task length can be customized.	+
Babyscreen	2014	Ireland, United Kingdom. Qatar, Gambia	English, French, Arabic, Swedish, German, Chinese, Spanish	18–36 month	Yes	Up to 20 min	+
BENCI	2010	Spain, Kenya, Morocco, Ecuador, Chile, Palestine, Argentine	Spanish, Arabic, Portuguese^, Chinese^, English	6–18 years	Yes	60–90 min [5–10 min per test]	++
DEEP	2017	India, Malawi	Hindi, Chichewa	2–6 years	Yes	30–45 min [2–4 min per test]	+++
Early Years Toolbox	2015	Numerous	English, Afrikaans, Chinese (Simplified and Traditional), Danish, Finnish, French, Indonesian, Japanese, Korean, Mongolian, Norwegian, Portuguese, Shangaan, Sotho, Spanish, Swedish, Xhosa, Zulu, and more in progress	3–6 years	Yes	25–45 min total [5–8 min per test]	+
ENABLE	2013	United Kingdom, Malawi, Brazil	English and Portuguese	6–8 years	Yes	30 min [4–6 minutes per test]	+
NeuroScreen	2012	United States, South Africa, Thailand, Kenya, Uganda, Senegal	English (multiple accents), Xhosa, Zulu, Afrikaans, Thai, Luganda, Luo, French, Swahili	13–65 years	Yes	25 min to complete 12 original/core subtests; customizable batteries of variable length up to 16 subtests total	+
Plus EF	2013	United States, Pakistan, Kenya	English, Sindhi, Swahili	8–12§ years	Yes	30 min [5–15 min per test]	+
RACER	2019	Lebanon (Syrian refugees), Niger, Peru, Ghana, Ethiopia, Bangladesh Indonesia	Multiple	4–90 years	Yes	15–40 min, [3–10 min per test]	+
START	2017	India	Hindi, English, Italian	2.5–7 years	In progress	30–45 min [5–9 min per test]	++
Tangerine EF Touch	2005	Kenya	English, Swahili	3–5 years	Yes	20–30 min [2–6 min per test]	+
Commercial tools							
APNA	2020	United States	English, Spanish	5–18 years	In progress	3–12 min per test	++
CANTAB	1980s	Numerous	>45 languages and dialects	4–90 years	Yes	2–18 min per test	+
Cogstate	1999	Numerous	>100 languages and dialects	6–99 years	Yes	2–11 min per test	+
MEFS/EFgo	2014	Numerous	English, Spanish, Mandarin, Hmong, Somali, Arabic, Turkish, Vietnamese and more (15 total)	2–70+ years	Yes	2–7 min	+
NIH Toolbox	2006	Numerous	English, Spanish, Cebuano, Swahili, Dholuo, Arabic (Saudi Arabia), Arabic (Egypt), French, Italian, Twi	3–85 years	Yes	5–8 min per test	++

* Type of validity testing varies by test and language. Updated as of September 2021. Please refer to S1 File for specific psychometric properties or contact the developers for more information.

^^^ Under development, at time of publication.

§ The Kenyan adaptation is used for 5–8 year olds.

† Training: All individuals require capacity for uniform test administration.

^+^Indicates <4 h of required training to perform.

^++^Indicates an estimated 4–12 h of required training.

^+++^Indicates an estimated >12 h of required training.

AMES, Assessment of Effort, Motivation, and Self-Regulation; APNA, Adaptable Pediatric Neurocognitive Assessment; BENCI, Batería de Evaluación Neuropsicológica Computerizada Infantil (English translation: Computerized Neuropsychological Battery for Children); CANTAB, Cambridge Neuropsychological Test Automated Battery; DEEP, DEvelopmental Assessment on an E-Platform; EF, Executive Functioning; ENABLE, Educational Neuroscience App-Based Learning Environment; MEFS, Minnesota Executive Function Scale; NIH, National Institutes of Health; PLUS EF, Promoting Learning, Understanding Self-Regulation Executive Functioning; RACER, Rapid Assessment of Cognitive and Emotional Regulation; START, Screening Tools for Autism Risk using Technology.

While nearly all commercial tools have age ranges that extend into adulthood, the non-commercial tools tend to have more narrow ranges, likely due to a target study population for whom the initial tool was developed. Most assessment tools contain batteries that consist of individual tests, and the length of the assessment is modifiable based on the number of tests selected. For example, Cantab and Cogstate each have over 16 tests that can be administered, depending on the needs of the user [33,34]. Most tools contain tests that each evaluate specific domains of cognition, often focusing on memory, attention, visual-spatial, and inhibition tasks (Table 2). Babyscreen and the Minnesota Executive Function Scale (MEFS)/EFgo test the youngest age range of children (as young as 18 months of age), but only have 1 test administered measuring multiple sub-domains of cognition [35,36]. The MEFS/Efgo tool administers a test based similar to the Dimensional Change Card Sort task, aiming to measure inhibition, cognitive flexibility, and memory [36]. This approach is linked to the challenges of tapping into specific, differentiated dimensions of cognitive tasks within these early ages [37].

**Table 2:** Domains measured by tablet-based cognitive assessments.

Tool name	Inhibition	Cognitive flexibility	Memory	Processing speed	Attention	Learning	Visual-spatial
Non-commercial							
AMES	+	+	+	+			
Babyscreen		+			+	+	
BENCI	+	+	+	+	+	+	+
DEEP	+	+	+	+	+		+
Early Years Toolbox	+	+	+			+	+
ENABLE			+	+	+	+	+
NeuroScreen	+	+	+	+	+	+	+
PLUS EF	+	+	+				
RACER	+		+			+	
START							+
Tangerine EF Touch	+	+	+	+	+		
Commercial tools							
APNA	+		+	+	+	+	+
CANTAB	+	+	+	+	+	+	+
Cogstate			+	+	+	+	+
MEFS/EFgo	+	+	+				
NIH Toolbox	+	+	+	+	+	+	

AMES, Assessment of Effort, Motivation, and Self-Regulation; APNA, Adaptable Pediatric Neurocognitive Assessment; BENCI, Batería de Evaluación Neuropsicológica Computerizada Infantil (English translation: Computerized Neuropsychological Battery for Children); CANTAB, Cambridge Neuropsychological Test Automated Battery; DEEP, DEvelopmental Assessment on an E-Platform; EF, Executive Functioning; ENABLE, Educational Neuroscience App-Based Learning Environment; MEFS, Minnesota Executive Functioning NIH-National Institutes of Health; PLUS EF, Promoting Learning, Understanding Self-Regulation Executive Functioning; RACER, Rapid Assessment of Cognitive and Emotional Regulation; START, Screening Tools for Autism Risk using Technology.

## 4. Considerations for identifying the tool(s) of choice

While the concept and aims of these tablet-based cognitive assessment tools are often similar, their strengths and limitations are highlighted for potential users to consider while identifying an appropriate tool for their setting. Some limitations are inherent to the way tools are funded and designed. Non-commercial tools are often developed by academicians with grant funding. These are therefore typically dependent on this funding or other program fees to maintain and update applications along with the tablet operating system. While these tools are often open-source, and hence costs are generally low, researchers often must directly contact developers to arrange for an agreement for tool use. While this benefits smaller projects with budgetary constraints, the lack of available support can cause delays in their implementation or any adjustments required.

Specific tools also have their own strengths and limitations. The Early Years Toolbox is the only tool that is freely downloadable and has instructions on administration on its website, a great strength in its accessibility. However, due to the “open access” nature of the tool, this results in limited knowledge on the full extent on locations of use and the degree of success of cross-cultural administration. This can only be gleaned from published articles and reports of its use, which may be subject to publication bias. A summary of the strengths and limitations for each tool is included in Table 3. Associated psychometrics and additional information about these tools are detailed in the S1 File.

**Table 3:** General strengths and limitations of tablet-based cognitive assessments.

Tool name	Strengths	Limitations
Non-commercial		
AMES	❖ Builds off of testing developed from PLUS EF ❖ Minimal training required to administer	❖ Validity testing is currently underway ❖ Need to contact developer directly to arrange an agreement for use of tool
Babyscreen	❖ Ability to use in children as young as 18 months ❖ Brief ❖ Minimal training required to administer ❖ Requires only 1 sentence to be translated in other languages at the start of the administration, with 7 language translations available	❖ Narrow age range for administration ❖ Only recommended for screening at this time ❖ Need to contact developer directly to arrange an agreement for use of tool
BENCI	❖ Has wide range of use for school-aged children ❖ Developers committed to providing access at no-cost	❖ Does not measure children under 6 years ❖ Lengthier administration (1 h+) ❖ Requires more than minimal training to administer ❖ Need to contact developer directly to arrange an agreement for use of tool
DEEP	❖ Minimal/no language requirements for administration of the application	❖ Requires additional training to administer ❖ Need to contact developer directly to arrange an agreement for use of tool
Early Years Toolbox	❖ Freely available and ready to download from tablet app store without direct consultation with developers ❖ Focused development team available for additional support and customized use for a nominal fee ❖ Minimal training required to administer; training materials are available on website ❖ Developers committed to providing access at no-cost	❖ Fairly narrow age range ❖ Known use and implementation is mostly limited to published studies, as the developers are not engaged in every use ❖ Minimal costing and support required for new language development or uploading data into a server
ENABLE	❖ Minimal training required to administer	❖ Narrow age range for administration ❖ Need to contact developer directly to arrange an agreement for use of tool
NeuroScreen	❖ Minimal training required to administer ❖ Robust language translation and adaptation methods used ❖ Some normative data available for adolescents	❖ Need to contact developer directly to arrange an agreement for use of tool ❖ Does not measure children under 13 years
PLUS EF	❖ Minimal training required to administer	❖ Need to contact developer directly to arrange an agreement for use of tool
RACER	❖ Large age range for testing, including adulthood ❖ Minimal training required to administer	❖ Does not have a dedicated technological developer team at this time, limiting support on use ❖ Need to contact creator directly to arrange an agreement for use of tool
START	❖ Minimal language requirements for administration of the application ❖ Also offers additional social domain testing, taking parent-child interaction videos for human analysis	❖ Validity testing is currently underway ❖ Requires additional training to administer ❖ Need to contact developer directly to arrange an agreement for use of tool
Tangerine EF Touch	❖ Strong validity data and prior use in Kenya ❖ Minimal training required to administer	❖ Requires a subscription to the Tangerine platform for use of EF touch ❖ Most international work has been focused only in Kenya ❖ Fairly narrow age range
Commercial Tools		
APNA	❖ NIH Funded Tool ❖ To be designed for languages and images to be adaptable to different populations	❖ Validity testing is currently underway; not yet available for use in 2021 ❖ Proprietary, cost unknown at this time ❖ Need to contact developer directly to arrange an agreement for use of tool ❖ Requires additional training to administer
CANTAB	❖ Large age range for testing, including adulthood (with data comparable across age ranges) ❖ Minimal training required to administer; training materials are available on website	❖ Proprietary, cost ❖ Pricing requires quotes from company ❖ Images within tests are not geared towards children, specifically
Cogstate	❖ Large age range for testing, including adulthood (with data comparable across age ranges) ❖ Minimal training required to administer; training materials are available on website	❖ Proprietary, cost ❖ Pricing requires quotes from company
MEFS/EFgo	❖ Initially an NIH Funded tool ❖ Has 2 different versions of the tools, 1 for research purposes with minimal changes, and another for educational purposes	❖ Proprietary, cost ❖ Only consists of 1 card-sorting task that measures 3 different domains of executive functioning
NIH Toolbox	❖ Large age range for testing, including adulthood (with data comparable across age ranges) ❖ NIH Funded Tool ❖ Pricing is standard for all assessments within the program within a subscription model, which can be used with up to 10 iPads ❖ Training materials are available on website	❖ Proprietary, cost ❖ Requires specific models of iPads and a Bluetooth keyboard ❖ Requires different subscriptions for different languages of assessments ❖ Images within tests are not geared towards children, specifically

AMES, Assessment of Effort, Motivation, and Self-Regulation; APNA, Adaptable Pediatric Neurocognitive Assessment; BENCI, Batería de Evaluación Neuropsicológica Computerizada Infantil (English translation: Computerized Neuropsychological Battery for Children); CANTAB, Cambridge Neuropsychological Test Automated Battery; DEEP, DEvelopmental Assessment on an E-Platform; EF, Executive Functioning; ENABLE, Educational Neuroscience App-Based Learning Environment; MEFS, Minnesota Executive Function Scale; NIH, National Institutes of Health; PLUS EF, Promoting Learning, Understanding Self-Regulation Executive Functioning; RACER, Rapid Assessment of Cognitive and Emotional Regulation; START, Screening Tools for Autism Risk using Technology.

We identified a number of factors we consider important when choosing a tool for use in a low-resourced setting for measuring cognition in early childhood. These include (a) ease of using technology to measure cognition in the early childhood; (b) validity in varying cultural contexts; (c) ease of adaptability for different settings; (d) overcoming workforce challenges; and (e) accessibility.

### Use of technology to assess cognition in the early childhood

There is still debate as to whether young children can be assessed appropriately and reliably using technology in the early years. Within this review, most tablet-based tools start testing at 3 years and older. In high-resourced settings, evidence suggests that children as young as 2 years may appropriately engage with digital technologies [38,39]. However, at these early ages, the foundations of executive functioning and cognition are being laid, adding to the challenge of isolating those constructs for testing [40]. Measurement of cognition at under 3 years of age may therefore, still be best performed with in-person or parent-report assessment tools [41] or with neurophysiological tests or imaging that map neural correlates underlying cognitive functions [42]. Many tools, especially non-commercial tools, are gamified, featuring appealing and narrative graphics for children. While this aids in overcoming the challenge of engaging children in assessment tools, the bright and interesting images may inadvertently lead to an overestimation of the domain of attention and impulsivity [27].

### Validity of tools for varying socioeconomic and cultural settings

**Fig 2:**
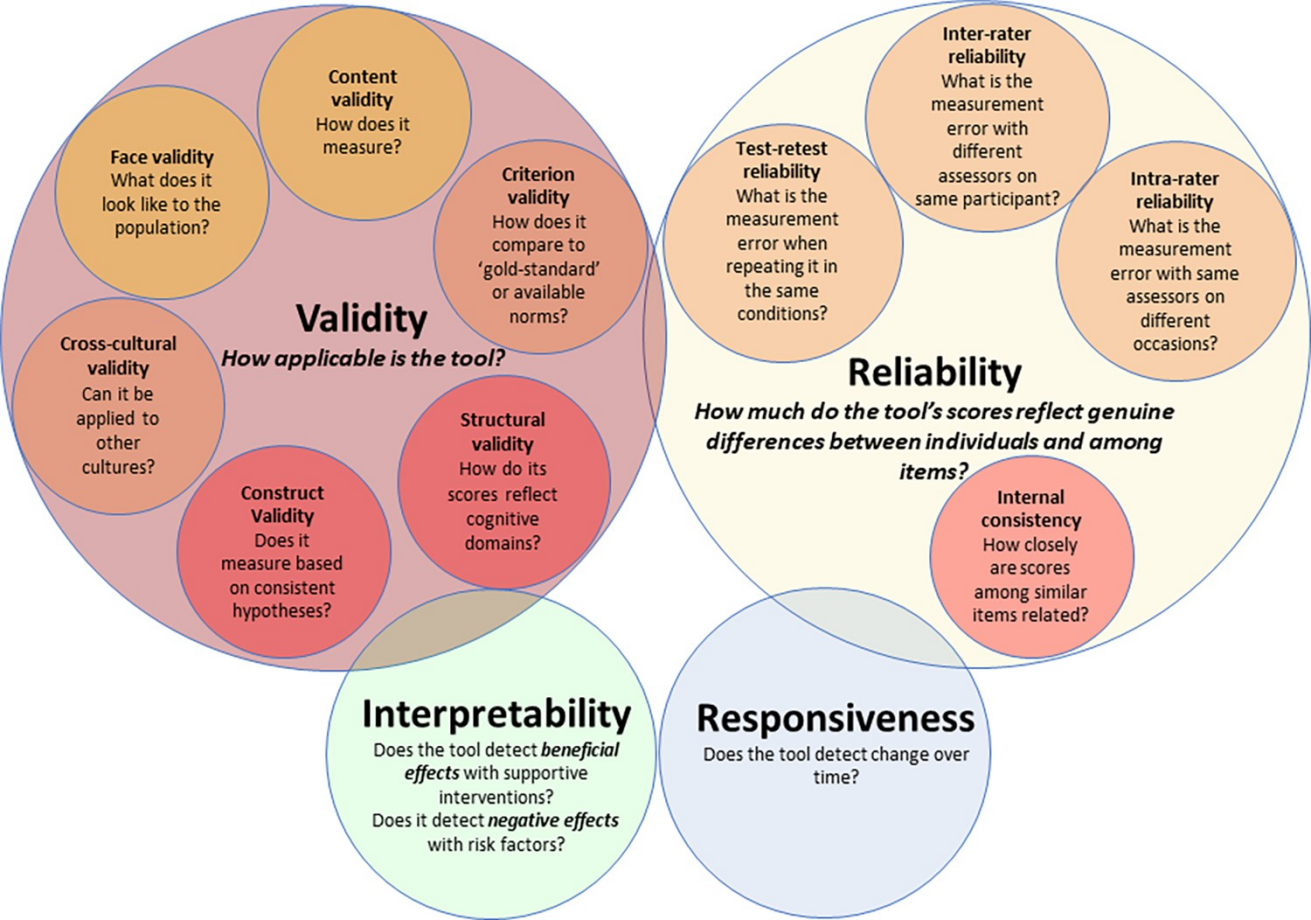
Assessing the measurement properties of a tool. Domains of psychometric properties adapted from the following publication: Mokkink LB, Terwee CB, Knol DL, Stratford PW, Alonso J, Patrick DL, et al. The COSMIN checklist for evaluating the methodological quality of studies on measurement properties: A clarification of its content. BMC Medical Research Methodology. 2010;10(1):22.

An important first step in choosing a cognitive tool is to critically review its measurement properties (such as validity and reliability). The COnsensus-based Standards for the selection of health Measurement INstruments (COSMIN) guidelines provide a framework to guide this [43] and supports the use of both qualitative (acceptability, face, and content validity) and quantitative aspects of validity (construct, cultural, and structural validity) alongside reliability (Fig 2). While nearly all the tablet-based cognitive tests included in this review (14/16) have some preliminary or published validity data (S1 File), the detail and rigor of this psychometric testing differs among tools. Some tools have attempted validation against a “gold standard.” However, this is complex, particularly in international low-resourced settings since “gold standard assessment tools” are typically developed and validated in high-resourced western settings.

Evaluating validity of tools can be particularly challenging within settings where children have limited daily exposure to technology or the items contained within the assessment tool. Tools are often developed for children with high exposure to technology, indicating that limited exposure may impact a child’s ability to interact with the tool’s content in the same meaningful way. However, evidence is emerging that suggests the lack of prior exposure to smart devices may not impact the accuracy with which tablet-based tools can measure cognition [28]. Indeed, a “rights-based perspective” would argue that technology coupled with culturally neutral content can benefit children globally. However, our review has identified rigorous psychometric testing as a gap in this field, which warrants additional consideration as tablet-based tools are disseminated widely.

Notably, caution should be taken when interpreting the assessment scores for children who differ significantly, either by language, age, or culture, from the original normative population of the assessment tool. Certain aspects of cognition are more adaptive and highly prioritized in some settings compared to others, and thus, the normative range of scores is likely to differ across settings. Thus, issues will arise when one depends on the normative population data to determine cutoffs for deficits in cognition or to function as a comparison or control group.

### Ease of adaptability of tools for different settings

The first generation of tablet-based assessment tools were programmed with a single language in mind, with no ability to adapt for different contexts or languages. More recent tools have built-in functionality to allow for easy adaptability between contexts and languages, as long as the appropriate adaptation methodologies are used. Some tools require very little language for their use. For example, Developmental Assessment on an E-Platform (DEEP) and Babyscreen have little to no language incorporated into the application, making linguistic adaptation an easy task [32,35]. Other tools contain more use of language, resulting in time-intensive adaptation processes involving forward and backward translations and cognitive interviewing to ensure face validity. This process takes multiple cycles and may require full re-programming of the test by assessment tool developers, the case for a recent cultural and linguistic adaptation of the NIH Toolbox for Kenya [44]. Even within a single country, certain images may need to be adjusted to accommodate the broadest range of cultures and contexts with the most familiar images used among cultures within the region.

### Overcoming workforce challenges

A significant advantage of tablet-based cognitive assessment tools is their ability to simultaneously administer, score, and record, either within the tablet computer itself, or a cloud server. By removing the requirement for a psychologist or highly trained individual to administer the tool, these assessment tools can be administered by non-specialists on a scale far beyond traditional psychological tests. Most of the non-commercial (8/11) and commercial (4/5) tools require only a few hours of training for test administration, allowing for assessors from a broad array of educational and training backgrounds. Nearly all the tests do not require an assessor’s observations as part of the scoring. One of the few exceptions is the NIH Toolbox’s List Sorting task, which measures working memory and requires an assessor to input whether a verbally repeated series of words is correct or incorrect [45]. With minimal input from the assessors, these tablet-administered tools reduce the risk of bias and human error and make great strides in overcoming workforce challenges in low-resourced settings.

### Accessibility

While few assessment tools are intended for commercial use, most have been created within academic settings and are freely available with open-source coding (Table 1). Developers of some tools, e.g., the BENCI and Early Years Toolbox, stipulate at inception that their tools remain free of cost so that they could be easily utilized in low-resourced settings. The few academic, non-commercial tools that require some funding directly relate the costs to the time required to support the developer in adapting the code for a new setting and other factors, such as server maintenance.

For commercial tools, some have a published fee for their use. This can range from a few hundred dollars for a subscription, to many thousand dollars when paying for tests per participant. Costs may vary dependent on the number of tests and administrations included within the assessment battery. While costs associated with the use of commercial assessment tools may be a deterrent for many researchers in low-resourced settings, it ensures tools used are adequately maintained, with up-to-date information technology infrastructure and developers on staff to troubleshoot any data-related issues.

## 5. Future directions for tablet-based pediatric cognitive assessment tools

This review aimed to summarize the current landscape of tablet-based assessment tools that measure cognition in children, particularly those used in low-resourced settings, as a potential solution to poor healthcare infrastructure and workforce-related barriers. We identified 16 tools that ranged the full spectrum of possibilities: from open-sourced to proprietary, and from those in their early stages of piloting in one region to those with extensive validation in multiple countries. As this novel area of digital pediatric cognitive assessment tools emerge and are pushed up the global mHealth agenda [29,46], it is critical for users to consider a tool’s psychometric properties, such as validity and reliability, before integrating it into clinical practice, research, or public health developmental surveillance systems. The COSMIN checklist provides useful guidance, not only to evaluate the validity and utility of a novel digital assessment tool, but also for developers to keep in mind while planning validation studies, or when describing the strengths and limitations of their tools to potential users [43].

In the absence of a true “gold-standard,” tool developers should aim to generate local normative scores for these novel tools in the target populations, instead of benchmarking them to the available “gold-standards.” The developers of the ENABLE and DEEP tool hope to develop a cloud database from their users, so that global sample “norms” can become available in open-source platforms (verbal communication from N. Pitchford and S. Bhavnani, respectively (February 2021)).

Additionally, while this review focuses on tablet-based assessment tools, we are keenly aware that smartphones represent the ultimate game changer in terms of achieving scale. The use of tablet-based assessment tools still requires a trained individual to go into the community or household for administration, whereas a smartphone-based assessment tool could potentially be downloaded onto a phone and then self-administered or administered by a parent within the home. Lead investigators of the NIH Toolbox are involved with the development of self-administered smartphone-based tests, Mobile Toolbox, which they hope will be available for public dissemination in 2023 [47]. Given the ubiquity of smartphones globally—over 5 billion subscribers, 70% of those residing in low-resourced settings—and cellular networks connecting 85% of the world’s population [29], we believe the potential for these cognitive assessment tools to scale will vastly improve with smartphones administration. Tablet-based tools were the focus on this review as there are currently a number of well-studied and validated options available for use in low-resources settings. They also form a useful “bridge” between more expensive and potentially fragile laptop computers and a larger screen than mobile devices. It is clear, however, that smartphones are more accessible and cheaper. Presently, less information is available about applications on these devices for measuring cognition in children, smartphone-based cognitive assessments for other populations, such as adults with dementia, have recently been developed [48,49]. It is likely that this review will need updating considering the fast-moving field and will need to include a focus on smartphone-based tools once data on their validity are available. Over 80% of World Health Organization (WHO) member states use at least 1 mHealth initiative operationalized through smartphones [29], with features such as the use of videos and decision support systems, that have proven useful in improving maternal and child health in a variety of low-resourced settings [50]. Therefore, the integration of cognitive assessments in smartphones may be the next leap towards optimizing child development at scale across global settings.

The digital administration of these tools also makes it possible to sync data collected from different modalities, such as eye tracking and electroencephalography, to provide a deeper, more integrated level of evaluation. With advances in technology, further spurred by substantial increases in use of telemedicine during the Coronavirus Disease 2019 (COVID-19) pandemic, these assessment tools may facilitate the possibility of virtual, parent-led, in-home well-baby checks in the future [51].

A forum to house the available digital tools, regularly updated to reflect the latest progress and associated data, would be valuable to stakeholders with interest in early childhood development. Such an effort has been initiated by the World Bank [52]. For this review, we aim to use an example of this and consolidate the information gleaned within the IMPACT Measures Tool database [53]. This online and open-source database is based on a research-driven scoring system that allows for the comparison of early childhood and parenting measures based on 4 categories (i.e., cost, usability, cultural relevance, and technical merit) [54]. Attending to the current landscape and new demands in low-resourced settings, we thought that the IMPACT database would benefit from the addition of digital measures, but other digital databases, as they emerge, should be considered.

This review has some limitations. Because we did not perform a formal systematic review, it is possible that we have missed some existing literature regarding tools currently being used in low-resourced settings outside of the network of investigators with whom we connected. Further, the dynamic and fast-paced nature of this emerging field would imply that new and updated tools are being added to the existing pool rapidly. Some have not yet been used in low-resourced settings and therefore will have not been included in this review. However, despite these limitations, we believe that this review of the existing tablet-based cognitive assessment tools adds great value, since the current landscape of available tools and possible future directions have not yet been summarized in this field.

### Brief considerations for developers of digital cognitive evaluation tools

While this review is primarily targeted for users of tablet-based tools measuring cognition, we provide the following recommendations for tool developers. A primary consideration in the development of new tools should be their scalability. To benefit the large number of children who are faltering in cognition development within low-resourced settings, these tools must have the ability to adopt at scale within clinical practice and health systems. The mHealth Assessment and Planning for Scale (MAPS) toolkit published by the WHO provides a useful guide for iterative assessment of tool readiness for scaling up of mHealth tools, as well as providing strategies to address common barriers inherent in the pathway to scaling up [54]. Similarly, “Beyond Scale” launched by the Digital Impact Alliance is a free online course that highlights key challenges and solutions to scaling up mHealth solutions [55].

While understanding the importance of scalability, we also strongly recommend that tool developers closely collaborate with stakeholders from low-resourced settings—children and their families, researchers, health system staff, and policy makers—across all phases of development to ensure that the tool is designed optimally for its intended contexts, is affordable for scale-up, and able to be integrated within the health and educational sectors [56]. In partnership with key stakeholders, developers should also consider the local laws about data privacy and security. Most of the tools described in this review collect only de-identified study IDs, with password-protected storage on secure servers and varying levels of encryption and password keys. Ensuring security of data within the tablets and cloud storage is an essential feature for this technology.

And finally, cognitive tools only have the potential for meaningful impact when they are disseminated and being utilized. To ensure that information is readily available to others, we recognize that the addition of digital assessment tools in online repositories of child development measures is essential [53,57]. These efforts will help researchers and practitioners to understand and be informed as to what is best for them to use when selecting evaluation tools for their programs in this fast moving and dynamic field.

### Conclusions

Tablet-based cognitive assessment tools may finally overcome the barriers of inadequate health systems that lead to poor measurement of child development outcomes in low-resourced settings. Data derived from these tools can then provide the foundation for drafting contextually relevant policies and practices—from the sub-local to the global—to optimize the developmental potential of all children globally.

## Supporting information

S1 File This supplement contains 2 appendices. Appendix A is a table outlining the psychometric measurement properties of the tablet-based cognitive tools included in this review. Appendix B is another table that describes the additional neurodevelopmental domains evaluated by tablet-based assessments, with contact information of the developers.
(DOCX)
