# Supplementary material for: The current landscape and future of tablet-based cognitive assessments for children in low-resourced settings
Source: PLOS Digit Health. 2023 Feb 23;2(2):e0000196. doi: 10.1371/journal.pdig.0000196 (PMC9949664; doi:10.1371/journal.pdig.0000196)
Supplement: S1 File — Appendix A is a table outlining the psychometric measurement properties of the tablet-based cognitive tools included in this review. Appendix B is another table that describes the additional neurodevelopmental domains evaluated by tablet-based assessments, with contact information of the developers. (DOCX) [file pdig.0000196.s001.docx]

Appendix A: Measurement Properties of Tablet-based Cognitive Tools, updated November 2022

| **Domain** | | **Measurement Property** | **Definition** | **AMES** | **Baby Screen**  **^1,2^** | **BENCI**  **^3-5^** | **DEEP**  **^6-9^** | **Early Years**  **Toolbox**  **^10-19^** | **ENABLE**  **^20-22^** | **NeuroScreen ^23-25^** | **Plus EF**  **^26-33^** | **RACER**  **^34-37^** | **START ^38^** | **Tangerine EF Touch**  **^39-47^** | **APNA** | **CANTAB**  **^48-57^** | **COG-STATE**  **^58-67^** | **MEFS/**  **EFgo^68,69^** | **NIH Toolbox**  **^70-79^** |
| --- | --- | --- | --- | --- | --- | --- | --- | --- | --- | --- | --- | --- | --- | --- | --- | --- | --- | --- | --- |
| Validity | | Face validity | The degree to which the items of a tool appear to be suitable for measurement, using local representatives from the study population. This is different to content or cross-cultural validity | - | + | +++ (Arabic version)  ++ (other versions) | +++ | +++ | +++ | ++ | ++ | +++ | ++ | +++ | - | +++ | ^ | ++ | + |
|  |  | Content validity | The degree to which a tool is an adequate reflection of the construct being measured.  The tools have been evaluated by expert review, including usage in low-resourced settings. | - | + | - | +++ | +++ | - | ++ | ++ | +++ | ++ | +++ | - | +++ | - | ++ | +++ |
|  |  | Construct validity | The degree to which the scores of a tool are consistent with hypotheses based on the assumption that the tool validly measures the construct to be measured.  Examples of this validity:   - Discriminant validity- testing whether concept or measurements that are not supposed to be related are actually unrelated. - Convergent validity with other measures (e.g. school results or other neurocognitive tools) or datasets from settings. | In progress | + | +++ (Arabic version)  ++ (Ecuadorian version) | In progress | +++ | +++ | +++ | ++ | +++ | ++ | +++ | In progress | +++ | +++ | + | + |
|  |  | Cross-cultural validity | Whether measures (in most cases psychological constructs) that were originally generated in a **single** culture are applicable, meaningful, and thus equivalent to be applied in **another** culture | - | In progress | +++ | In progress | +++ | +++ | +++ | In progress | +++ | In progress | +++ | - | +++ | - | ++ | +++ |
|  |  | Criterion validity | This is the degree to which the scores of a tool are an adequate reflection of a ‘gold standard’ test. This is challenging, as no single ‘gold standard’ child neurodevelopment measurement, partly due to its context-specific nature.  For individual tools, most normative values come from high-income settings. If available, the possibility of complementary datasets on similar tests from children in low-resourced settings can assist with this. | - | + | - | +++ | +++ | +++ | +++ | + | - | In progress | +++ | In progress | +++ | ^ | ++ | + |
|  |  | Structural validity | The degree to which the scores of a tool are an adequate reflection of the dimensionality of the construct to be measured (with the use of psychometric analysis such as structural equation modeling). | In progress | - | +++ (Arabic version) | In progress | +++ | - | ++ | + | - | In progress | ++ | - | + | +++ | - | + |
| Reliability | | Reliability | The proportion of the total variance in the measurements due to ‘true’ differences between children, by including aspects of:   - Test re-test (performing the same test twice or more within a brief period)   - Different assessors at the same occasion (inter-rater reliability)   - Same assessors at different occasions (intra-rater reliability) | - | - | +++ (Arabic version)  ++ (Ecuadorian and Kenyan versions) | In progress | ++ | +++ | ++ | + | - | In progress | + | - | +++ | +++ | + | + |
|  |  | Internal Consistency | This is the degree of interrelatedness among the items | In progress | + | - | In progress | +++ | +++ | ++ | n/a | n/a | In progress | +++ | - | +++ | - | - | + |
| Interpretability | | Known-Groups Validity | Deleterious effects may be demonstrated with known risk factors such as child stunting, HIV exposure or poverty ^80^  Beneficial effects may be demonstrated with nutrition or other supportive interventions ^80^ | - | + | ++ (Kenyan and Arabic versions) | ++ | +++ | - | +++ | + | +++ | In progress | + | - | +++ | +++ | ++ | + |
| Responsiveness | | Responsiveness | The ability of an outcome measure to detect change over time in the construct to be measured (e.g. typically improvement with increasing age). | - | + | +++ | In progress | +++ | - | - | + | - | In progress | +++ | - | +++ | + | + | + |
|  | Notes:  +++: Has been performed within a low-resourced setting and data are published in a peer-reviewed journal  ++: Has been performed within a low-resourced setting but the data are not (yet) published in a peer-reviewed journal  +: Has not been performed yet within a low-resourced setting but has been published in a high-resourced setting  -: Has not been performed yet  ^: Performed in adults, but not in pediatric populations.  **Abbreviations:** *AMES- Assessment of Effort, Motivation, and Self-Regulation; APNA- Adaptable Pediatric Neurocognitive Assessment; BENCI- Batería de Evaluación Neuropsicológica Computerizada Infantil (English translation: Computerized Neuropsychological Battery for Children); CANTAB- Cambridge Neuropsychological Test Automated Battery; DEEP – DEvelopmental Assessment on an E-Platform; EF- Executive Functioning; ENABLE- Educational Neuroscience App-Based Learning Environment; MEFS- Minnesota Executive Function Scale; NIH-National Institutes of Health; PLUS EF- Promoting Learning, Understanding Self-Regulation- Executive Functioning; RACER- Rapid Assessment of Cognitive and Emotional Regulation; START – Screening Tools for Autism Risk using Technology*  Domains and definitions adapted from: Mokkink LB, Terwee CB, Patrick DL, Alonso J, Stratford PW, Knol DL, Bouter LM, de Vet HC. The COSMIN study reached international consensus on taxonomy, terminology, and definitions of measurement properties for health-related patient-reported outcomes. J Clin Epidemiol. 2010 Jul;63(7):737-45. doi: 10.1016/j.jclinepi.2010.02.006. | | | | | | | | | | | | | | | | | | |

References:

1. Twomey DM, Ahearne C, Hennessy E, et al. Concurrent validity of a touchscreen application to detect early cognitive delay. *Archives of Disease in Childhood.* 2020:archdischild-2019-318262.

2. Twomey DM, Wrigley C, Ahearne C, et al. Feasibility of using touch screen technology for early cognitive assessment in children. *Arch Dis Child.* 2018;103(9):853-858.

3. Burneo-Garcés C, Cruz-Quintana F, Pérez-García M, Fernández-Alcántara M, Fasfous A, Pérez-Marfil MN. Interaction between socioeconomic status and cognitive development in children aged 7, 9, and 11 years: A cross-sectional study. *Developmental Neuropsychology.* 2019;44(1):1-16.

4. Fasfous AF, Peralta-Ramirez MI, Pérez-Marfil MN, Cruz-Quintana F, Catena-Martinez A, Pérez-García M. Reliability and validity of the Arabic version of the computerized Battery for Neuropsychological Evaluation of Children (BENCI). *Child neuropsychology : a journal on normal and abnormal development in childhood and adolescence.* 2015;21(2):210-224.

5. Maina RW, Abubakar A, Miguel P-G, Van De Vijver FJR, Kumar M. Standardization of the Computerized Battery for Neuropsychological Evaluation of Children (BENCI) in an urban setting, in Kenya: a study protocol. *BMC Research Notes.* 2019;12(1):799.

6. Bhavnani S, Mukherjee D, Dasgupta J, et al. Development, feasibility and acceptability of a gamified cognitive DEvelopmental assessment on an E-Platform (DEEP) in rural Indian pre-schoolers - a pilot study. *Glob Health Action.* 2019;12(1):1548005.

7. Mukherjee D, Bhavnani S, Swaminathan A, et al. Proof of Concept of a Gamified DEvelopmental Assessment on an E-Platform (DEEP) Tool to Measure Cognitive Development in Rural Indian Preschool Children. *Frontiers in psychology.* 2020;11:1202-1202.

8. Bhavnani S, Mukherjee D, Bhopal S, et al. The association of a novel digital tool for assessment of early childhood cognitive development, &#x2018;DEvelopmental assessment on an E-Platform (DEEP)&#x2019;, with growth in rural India: A proof of concept study. *eClinicalMedicine.* 2021;37.

9. Mukherjee D, Bhopal S, Bhavnani S, et al. The effect of cumulative early life adversities, and their differential mediation through hair cortisol levels, on childhood growth and cognition: Three-year follow-up of a birth cohort in rural India. *Wellcome open research.* 2022;7:74.

10. Cook CJ, Howard SJ, Scerif G, et al. Associations of physical activity and gross motor skills with executive function in preschool children from low‐income South African settings. *Developmental science.* 2019;22(5):e12820.

11. Howard SJ, Okely AD. Catching Fish and Avoiding Sharks: Investigating Factors That Influence Developmentally Appropriate Measurement of Preschoolers' Inhibitory Control. *J Psychoeduc Assess.* 2015;33(6):585-596.

12. Howard SJ, Cook CJ, Everts L, et al. Challenging socioeconomic status: A cross‐cultural comparison of early executive function. *Developmental science.* 2020;23(1):e12854.

13. McNeill J, Howard SJ, Vella SA, Cliff DP. Cross-Sectional Associations of Application Use and Media Program Viewing with Cognitive and Psychosocial Development in Preschoolers. *International journal of environmental research and public health.* 2021;18(4).

14. Howard SJ, Melhuish E. An early years toolbox for assessing early executive function, language, self-regulation, and social development: Validity, reliability, and preliminary norms. *Journal of Psychoeducational Assessment.* 2017;35(3):255-275.

15. Howard SJ, Powell T, Vasseleu E, Johnstone S, Melhuish E. Enhancing preschoolers’ executive functions through embedding cognitive activities in shared book reading. *Educational Psychology Review.* 2017;29(1):153-174.

16. Howard SJ, Vasseleu E. Self-Regulation and Executive Function Longitudinally Predict Advanced Learning in Preschool. *Frontiers in psychology.* 2020;11:49-49.

17. Martins CML, Bandeira PFR, Lemos N, et al. A Network Perspective on the Relationship between Screen Time, Executive Function, and Fundamental Motor Skills among Preschoolers. *International journal of environmental research and public health.* 2020;17(23).

18. Kuzik N, Naylor P-J, Spence JC, Carson V. Movement behaviours and physical, cognitive, and social-emotional development in preschool-aged children: Cross-sectional associations using compositional analyses. *PLOS ONE.* 2020;15(8):e0237945.

19. Berg V, Rogers SL, McMahon M, Garrett M, Manley D. A Novel Approach to Measure Executive Functions in Students: An Evaluation of Two Child-Friendly Apps. *Frontiers in psychology.* 2020;11:1702-1702.

20. Pitchford NJ, Outhwaite LA. Can Touch Screen Tablets be Used to Assess Cognitive and Motor Skills in Early Years Primary School Children? A Cross-Cultural Study. *Frontiers in Psychology.* 2016;7(1666).

21. Pitchford NJ, Chigeda A, Hubber PJ. Interactive apps prevent gender discrepancies in early-grade mathematics in a low-income country in sub-Sahara Africa. *Developmental science.* 2019;22(5):e12864.

22. Pitchford NJ, Kamchedzera E, Hubber PJ, Chigeda AL. Interactive Apps Promote Learning of Basic Mathematics in Children With Special Educational Needs and Disabilities. *Front Psychol.* 2018;9:262.

23. Robbins RN, Kluisza L, Liu J, et al. Construct Validity Supports Use of a Novel, Tablet-Based Neurocognitive Assessment for Adolescents and Young Adults Affected by Perinatal HIV from Vulnerable Communities in the United States. *AIDS and Behavior.* 2021;25(4):1185-1191.

24. Robbins RN, Santoro AF, Ferraris C, et al. Adaptation and construct validity evaluation of a tablet-based, short neuropsychological test battery for use with adolescents and young adults living with HIV in Thailand. *Neuropsychology.* 2022;36:695-708.

25. Katzef C, Henry M, Gouse H, Robbins RN, Thomas KGF. A culturally fair test of processing speed: Construct validity, preliminary normative data, and effects of HIV infection on performance in South African adults. *Neuropsychology.* 2019;33(5):685-700.

26. Obradović J, Sulik MJ, Finch JE, Tirado-Strayer N. Assessing students' executive functions in the classroom: Validating a scalable group-based procedure. *Journal of Applied Developmental Psychology.* 2018;55:4-13.

27. Finch JE, Obradović J. Independent and compensatory contributions of executive functions and challenge preference for students' adaptive classroom behaviors. *Learning and Individual Differences.* 2017;55:183-192.

28. Bardack S, Obradović J. Observing teachers' displays and scaffolding of executive functioning in the classroom context. *Journal of Applied Developmental Psychology.* 2019;62:205-219.

29. Sulik MJ, Obradović J. Teachers' rankings of children's executive functions: Validating a methodology for school-based data collection. *Journal of experimental child psychology.* 2018;173:136-154.

30. Garcia EB, Sulik MJ, Obradović J. Teachers’ perceptions of students’ executive functions: Disparities by gender, ethnicity, and ELL status. *Journal of Educational Psychology.* 2019;111(5):918-931.

31. Sulik MJ, Haft SL, Obradović J. Visual-Motor Integration, Executive Functions, and Academic Achievement: Concurrent and Longitudinal Relations in Late Elementary School. *Early Education and Development.* 2018;29(7):956-970.

32. Sulik MJ, Finch JE, Obradović J. Moving beyond executive functions: Challenge preference as a predictor of academic achievement in elementary school. *Journal of experimental child psychology.* 2020;198:104883.

33. Finch JE, Garcia EB, Sulik MJ, Obradović J. Peers Matter: Links Between Classmates’ and Individual Students’ Executive Functions in Elementary School. *AERA Open.* 2019;5(1):2332858419829438.

34. Ford CB, Kim HY, Brown L, Aber JL, Sheridan MA. A cognitive assessment tool designed for data collection in the field in low- and middle-income countries. *Research in Comparative and International Education.* 2019;14(1):141-157.

35. Chen A, Panter-Brick C, Hadfield K, Dajani R, Hamoudi A, Sheridan M. Minds Under Siege: Cognitive Signatures of Poverty and Trauma in Refugee and Non-Refugee Adolescents. *Child Development.* 2019;90(6):1856-1865.

36. Kim HY, Brown L, Tubbs Dolan C, Sheridan M, Aber JL. Post-migration risks, developmental processes, and learning among Syrian refugee children in Lebanon. *Journal of Applied Developmental Psychology.* 2020;69:101142.

37. Yuan H, Ocansey M, Sheridan M, et al. Validation of a Tablet-based Assessment Tool for Measuring Cognition among Children 4-6 Years of Age in Ghana. Under review by Internation Journal of Methods in Psychiatric Research2020.

38. Dubey I, Brett S, Ruta L, et al. Quantifying preference for social stimuli in young children using two tasks on a mobile platform. *PLOS ONE.* 2022;17(6):e0265587.

39. Willoughby MT, Wirth RJ, Blair CB, Family Life Project I. Executive function in early childhood: longitudinal measurement invariance and developmental change. *Psychological assessment.* 2012;24(2):418-431.

40. Magnus BE, Willoughby MT, Blair CB, Kuhn LJ. Integrating Item Accuracy and Reaction Time to Improve the Measurement of Inhibitory Control Abilities in Early Childhood. *Assessment.* 2019;26(7):1296-1306.

41. Willoughby MT, Blair CB, Wirth RJ, Greenberg M. The measurement of executive function at age 3 years: psychometric properties and criterion validity of a new battery of tasks. *Psychol Assess.* 2010;22(2):306-317.

42. Willoughby MT, Blair CB, Wirth RJ, Greenberg M. The measurement of executive function at age 5: psychometric properties and relationship to academic achievement. *Psychol Assess.* 2012;24(1):226-239.

43. Willoughby MT, Blair CB. Measuring executive function in early childhood: A case for formative measurement. *Psychol Assess.* 2016;28(3):319-330.

44. Willoughby MT, Piper B, Kwayumba D, McCune M. Measuring executive function skills in young children in Kenya. *Child neuropsychology : a journal on normal and abnormal development in childhood and adolescence.* 2019;25(4):425-444.

45. Willoughby MT, Piper B, Oyanga A, Merseth King K. Measuring executive function skills in young children in Kenya: Associations with school readiness. *Developmental science.* 2019;22(5):e12818.

46. Willoughby MT, Piper B, King KM, Nduku T, Henny C, Zimmermann S. Testing the Efficacy of the Red-Light Purple-Light Games in Preprimary Classrooms in Kenya. *Frontiers in Psychology.* 2021;12(668).

47. Willoughby MT, Kuhn LJ, Blair CB, Samek A, List JA. The test-retest reliability of the latent construct of executive function depends on whether tasks are represented as formative or reflective indicators. *Child neuropsychology : a journal on normal and abnormal development in childhood and adolescence.* 2017;23(7):822-837.

48. Cassidy AR. Executive function and psychosocial adjustment in healthy children and adolescents: A latent variable modelling investigation. *Child neuropsychology : a journal on normal and abnormal development in childhood and adolescence.* 2016;22(3):292-317.

49. Ismatullina V, Voronin I, Shelemetieva A, Malykh S. Cross-cultural Study of Working Memory in Adolescents. *Procedia - Social and Behavioral Sciences.* 2014;146:353-357.

50. Lee R, Singh L, van Liefde D, et al. Milk Powder Added to a School Meal Increases Cognitive Test Scores in Ghanaian Children. *The Journal of nutrition.* 2018;148(7):1177-1184.

51. Lelijveld N, Jalloh AA, Kampondeni SD, et al. Brain MRI and cognitive function seven years after surviving an episode of severe acute malnutrition in a cohort of Malawian children. *Public health nutrition.* 2019;22(8):1406-1414.

52. Mitra-Ganguli T, Kalita S, Bhushan S, et al. A Randomized, Double-Blind Study Assessing Changes in Cognitive Function in Indian School Children Receiving a Combination of Bacopa monnieri and Micronutrient Supplementation vs. Placebo. *Frontiers in pharmacology.* 2017;8:678.

53. Nkhoma OW, Duffy ME, Davidson PW, et al. Nutritional and cognitive status of entry-level primary school children in Zomba, rural Malawi. *International journal of food sciences and nutrition.* 2013;64(3):282-291.

54. Rose SA, Feldman JF, Jankowski JJ, Van Rossem R. Basic Information Processing Abilities at 11 years Account for Deficits in IQ Associated with Preterm Birth. *Intelligence.* 2011;39(4):198-209.

55. Toornstra A, Hurks PPM, Van der Elst W, Kok G, Curfs LMG. Measuring visual matching and short-term recognition memory with the CANTAB® Delayed Matching to Sample task in schoolchildren: Effects of demographic influences, multiple outcome measures and regression-based normative data. *Child Neuropsychology.* 2020;26(2):189-218.

56. Toornstra A, Hurks PPM, Van der Elst W, Massar K, Kok G, Curfs LMG. Measuring Goal Setting in School-Aged Children: Studying the Effects of Demographic Variables in Regression-Based Norms. *Journal of Pediatric Neuropsychology.* 2020;6(2):96-110.

57. Toornstra A, Hurks PPM, Van der Elst W, Kok G, Curfs LMG. Measuring Visual, Spatial, and Visual Spatial Short-Term Memory in Schoolchildren: Studying the Influence of Demographic Factors and Regression-Based Normative Data. *Journal of Pediatric Neuropsychology.* 2019;5(3):119-131.

58. Bangirana P, Sikorskii A, Giordani B, Nakasujja N, Boivin MJ. Validation of the CogState battery for rapid neurocognitive assessment in Ugandan school age children. *Child Adolesc Psychiatry Ment Health.* 2015;9:38-38.

59. Boivin MJ, Busman RA, Parikh SM, et al. A pilot study of the neuropsychological benefits of computerized cognitive rehabilitation in Ugandan children with HIV. *Neuropsychology.* 2010;24(5):667-673.

60. Chou CC, Pressler SJ, Giordani B, Fetzer SJ. Validation of the Chinese version of the CogState computerised cognitive assessment battery in Taiwanese patients with heart failure. *Journal of clinical nursing.* 2015;24(21-22):3147-3154.

61. Cromer JA, Schembri AJ, Harel BT, Maruff P. The nature and rate of cognitive maturation from late childhood to adulthood. *Frontiers in Psychology.* 2015;6(704).

62. de Jager CA, Schrijnemaekers AC, Honey TE, Budge MM. Detection of MCI in the clinic: evaluation of the sensitivity and specificity of a computerised test battery, the Hopkins Verbal Learning Test and the MMSE. *Age and ageing.* 2009;38(4):455-460.

63. Dingwall KM, Gray AO, McCarthy AR, Delima JF, Bowden SC. Exploring the reliability and acceptability of cognitive tests for Indigenous Australians: a pilot study. *BMC Psychology.* 2017;5(1):26.

64. Dingwall KM, Lewis MS, Maruff P, Cairney S. Reliability of repeated cognitive testing in healthy Indigenous Australian adolescents. *Australian Psychologist.* 2009;44(4):224-234.

65. Falleti MG, Maruff P, Collie A, Darby DG. Practice effects associated with the repeated assessment of cognitive function using the CogState battery at 10-minute, one week and one month test-retest intervals. *Journal of clinical and experimental neuropsychology.* 2006;28(7):1095-1112.

66. Yoshida T, Suga M, Arima K, et al. Criterion and Construct Validity of the CogState Schizophrenia Battery in Japanese Patients with Schizophrenia. *PLOS ONE.* 2011;6(5):e20469.

67. Zhong N, Jiang H, Wu J, et al. Reliability and Validity of the CogState Battery Chinese Language Version in Schizophrenia. *PLOS ONE.* 2013;8(9):e74258.

68. Beck DM, Schaefer C, Pang K, Carlson SM. Executive Function in Preschool Children: Test–Retest Reliability. *Journal of Cognition and Development.* 2011;12(2):169-193.

69. Hassinger-Das B, Jordan NC, Glutting J, Irwin C, Dyson N. Domain-general mediators of the relation between kindergarten number sense and first-grade mathematics achievement. *Journal of experimental child psychology.* 2014;118:78-92.

70. Casaletto KB, Umlauf A, Marquine M, et al. Demographically Corrected Normative Standards for the Spanish Language Version of the NIH Toolbox Cognition Battery. *J Int Neuropsychol Soc.* 2016;22(3):364-374.

71. Weintraub S, Bauer PJ, Zelazo PD, et al. I. NIH Toolbox Cognition Battery (CB): introduction and pediatric data. *Monogr Soc Res Child Dev.* 2013;78(4):1-15.

72. Zelazo PD, Anderson JE, Richler J, Wallner-Allen K, Beaumont JL, Weintraub S. II. NIH Toolbox Cognition Battery (CB): measuring executive function and attention. *Monogr Soc Res Child Dev.* 2013;78(4):16-33.

73. Bauer PJ, Dikmen SS, Heaton RK, Mungas D, Slotkin J, Beaumont JL. III. NIH Toolbox Cognition Battery (CB): measuring episodic memory. *Monogr Soc Res Child Dev.* 2013;78(4):34-48.

74. Gershon RC, Slotkin J, Manly JJ, et al. IV. NIH Toolbox Cognition Battery (CB): measuring language (vocabulary comprehension and reading decoding). *Monogr Soc Res Child Dev.* 2013;78(4):49-69.

75. Bauer PJ, Zelazo PD. IX. NIH Toolbox Cognition Battery (CB): summary, conclusions, and implications for cognitive development. *Monogr Soc Res Child Dev.* 2013;78(4):133-146.

76. Taylor BK, Frenzel MR, Eastman JA, et al. Reliability of the NIH toolbox cognitive battery in children and adolescents: a 3-year longitudinal examination. *Psychological medicine.* 2020:1-10.

77. Duffey M, Ayuku D, Ayodo G, et al. Translation and Cultural Adaptation of NIH Toolbox Cognitive Tests into Swahili and Dholuo Language for Use in Children in Western Kenya. *J Int Neuropsychol Soc.* 2021;IN PRESS, accepted 22 March 2021.

78. Mungas D, Widaman K, Zelazo PD, et al. VII. NIH Toolbox Cognition Battery (CB): factor structure for 3 to 15 year olds. *Monogr Soc Res Child Dev.* 2013;78(4):103-118.

79. Akshoomoff N, Beaumont JL, Bauer PJ, et al. VIII. NIH Toolbox Cognition Battery (CB): composite scores of crystallized, fluid, and overall cognition. *Monographs of the Society for Research in Child Development.* 2013;78(4):119-132.

80. Walker SP, Wachs TD, Grantham-McGregor S, et al. Inequality in early childhood: risk and protective factors for early child development. *The Lancet.* 2011;378(9799):1325-1338.

Appendix B: Additional Domains Evaluated by Tablet-Based Assessments, with Contact Information

| Tool name | Cognitive Domain | Test Name | Contact information for tool |
| --- | --- | --- | --- |
| Non-Commercial Tools | | | |
| AMES | Hearts and Flowers | inhibitory control and cognitive flexibility | The Obradovic Lab  [obradovic-lab@stanford.edu](mailto:obradovic-lab@stanford.edu)  https://sparklab.stanford.edu/ames |
|  | Spatial Memory | Short term spatial memory, spatial working memory |  |
|  | Color Dot | Processing speed |  |
| Babyscreen | Selective Attention | n/a | Deirdre Murray  [D.Murray@ucc.ie](mailto:D.Murray@ucc.ie) |
|  | Working Memory | n/a |  |
|  | Hidden object retrieval and object permanence | n/a |  |
|  | Learning | n/a |  |
| BENCI | Visual motor coordination | Trail-Making Task Part A | Miguel Perez Garcia:  mperezg@ugr.es |
|  | Memory and Learning | Episodic memory- visual and verbal recognition task |  |
|  | Executive function | Trail-Making Task Part B (animals, circles, rectangles)  Working memory  Go-no go  Spatial Stroop  Abstract Reasoning  Amusement Park |  |
|  | Attention | Continuous Performance Test (CPT, Sustained attention) and selective attention |  |
|  | Language | Verbal Comprehension |  |
|  | Language | Language Production |  |
|  | Language | Phonetic Fluency |  |
|  | Semantic Fluency | Semantic Fluency |  |
|  | Processing Speed | Reaction Time |  |
| DEEP | Manual Processing speed | Single Tap, Alternate Tap, Popping Bubbles | deep.support@sangath.in  Supriya Bhavnani:  [supriya.bhavnani@sangath.in](mailto:supriya.bhavnani@sangath.in)  Gauri Divan:  [gauri.divan@sangath.in](mailto:gauri.divan@sangath.in) |
|  | Manual coordination | Alternate Tap, Popping Bubbles |  |
|  | Response inhibition | Grow Your Garden |  |
|  | Divided attention | Hidden Objects |  |
|  | Reasoning | Odd One Out, Series Completion |  |
|  | Visual form perception | Matching Shapes, Spot the Difference, Pattern Making, Sorting Objects |  |
|  | Visual integration | Jigsaw, Spot the Difference, Pattern Making |  |
|  | Memory | Location Recall, Sequence Recall |  |
|  | Categorization | Sorting Objects |  |
| Early Years Toolbox | Visual-spatial working memory | Mr Ant | <http://www.eytoolbox.com.au> |
|  | Phonological working memory | Not This |  |
|  | Shifting | Card Sorting |  |
|  | Inhibition | Go/No Go |  |
|  | Vocabulary | Expressive Vocabulary |  |
|  | Early Numeracy | Early Numeracy |  |
| ENABLE | mathematical skills | n/a | Nicola Pitchford:  [Nicola.Pitchford@nottingham.ac.uk](mailto:Nicola.Pitchford@nottingham.ac.uk) |
|  | visual attention | n/a |  |
|  | short-term memory | n/a |  |
|  | manual processing speed | n/a |  |
|  | spatial intelligence | n/a |  |
|  | working memory | n/a |  |
|  | manual coordination | n/a |  |
| NeuroScreen | Learning | Verbal List Learning, Nonverbal Learning | [www.neuro-screen.com](http://www.neuro-screen.com)  Reuben N Robbins  [rnr2110@cumc.columbia.edu](mailto:rnr2110@cumc.columbia.edu) |
|  | Memory/Delayed Recall | Verbal List Learning Delayed Recall, Nonverbal Memory |  |
|  | Executive Functioning (cognitive flexibility, problem solving, inhibitory control) | Trail Making 2 (alternating colors and numbers), Mazes, Flanker |  |
|  | Processing Speed | Number Speed, Visual Discrimination 1 and 2, Trail Making 1 and 3 |  |
|  | Working Memory | Number Span, Nonverbal Working Memory |  |
|  | Motor Speed | Finger Tapping |  |
| Plus EF | Heart and Flowers | inhibitory control and cognitive flexibility | The Obradovic Lab  [obradovic-lab@stanford.edu](mailto:obradovic-lab@stanford.edu) |
|  | Multisource interference Test | inhibitory control |  |
|  | Flanker | inhibitory control |  |
|  | Digit Span Backwards | Working memory |  |
| RACER | Inhibitory Control | “Simon Task” or Spatial incompatibility task | Margaret Sheridan  [sheridan.margaret@unc.edu](mailto:sheridan.margaret@unc.edu) |
|  | Spatial Working Memory | Spatial Delayed Match to Sample |  |
|  | Episodic Memory | Paired Associate Learning |  |
|  | Implicit Learning | Serial Reaction Time |  |
| START | Social preference | Preferential Looking and Button task | Bhismadev Chakrabarti  [b.chakrabarti@reading.ac.uk](mailto:b.chakrabarti@reading.ac.uk) |
|  | Sensory sensitivity | Wheel task |  |
|  | Visuo-motor coordination | Butterfly task, bubble task, colouring task |  |
|  | Social communication | Parent-child interaction videos |  |
| Tangerine EF Touch | Simple Reaction | Bubble | Michael Willoughby:  [mwilloughby@rti.org](mailto:mwilloughby@rti.org) |
|  | Inhibitory Control | Silly Sounds Stroop |  |
|  | Inhibitory Control | Animal Go/No Go Task |  |
|  | Inhibitory Control and Cognitive Flexibility | Arrows Task |  |
|  | Working Memory | Pick the Picture |  |
|  | Attention Shifting and Flexible Thinking | Something is the Same |  |
| Commercial Tools | | | |
| APNA | Response Speed, Attention | Visual Motor Reaction Time | Odile Clavier: [ohc@creare.com](mailto:ohc@creare.com) |
|  | Strategic Learning, Memory Retention, Effort | Sort Picture Memory |  |
|  | Inhibition | Stop Signal |  |
|  | Response Speed, Attention Switching, Visual Search | Symbol Digit Modality Like |  |
|  | Working Memory, Sustained Attention | N-Back |  |
|  | Learning Acquisition, Memory Retention | Design Memory Forced Choice (Optional) |  |
|  | Cognitive Ability, Visual Reasoning | Odd One Out (Optional) |  |
| CANTAB | Visual episodic memory | Paired Associates Learning | <https://www.cambridgecognition.com/cantab/> |
|  | Working memory and executive functioning | Spatial Working Memory |  |
|  | Sustained attention | Rapid Visual Information Processing |  |
|  | Visuospatial working memory | Spatial Span |  |
|  | Visual recognition memory | Pattern Recognition Memory |  |
|  | Planning | Stockings of Cambridge |  |
|  | Emotion recognition | Emotional Recognition Task |  |
|  | Attention and short-term visual memory | Delayed Matching to Sample |  |
|  | Decision making | Cambridge Gambling Task |  |
|  | Attention set shifting | Intra/Extra Dimensional Set Shift |  |
|  | Emotional bias | Emotional Bias Task |  |
|  | Sensorimotor functioning & comprehension | Motor Screening Task |  |
|  | Processing & psychomotor speed | Reaction Time Test |  |
|  | Multitasking | Multi-Tasking Test |  |
|  | Response inhibition | Stop Signal Task |  |
|  | Verbal memory & new learning | Verbal Recognition Memory |  |
|  | Attention & visual search | Match to Sample Visual Search |  |
|  | Planning and executive function | One Touch Stockings of Cambridge |  |
| Cogstate | Memory | Behavioral Pattern Separation Object Test | <https://www.cogstate.com/> |
|  | Paired Associate Learning | Continuous Paired Associate Learning Test |  |
|  | Psychomotor Function | Detection Test |  |
|  | Memory | Face Name Associative Memory Exam |  |
|  | Upper Limb Motor Function | Finger Tapping Test |  |
|  | Visual Motor Control | Groton Maze Chase Test |  |
|  | Executive Function | Groton Maze Learning Test |  |
|  | Attention | Identification Test |  |
|  | Processing Speed | International Daily Symbol Substitution Test – Medicines |  |
|  | Processing Speed | International Digit Symbol Substitution Test – Symbols |  |
|  | Verbal Learning | International Shopping List Test |  |
|  | Working Memory | One Back Test |  |
|  | Visual Learning | One Card Learning Test |  |
|  | Emotional Recognition | Social-Emotional Cognition Test |  |
|  | Sustained Attention | Sustained Attention Test |  |
|  | Working Memory | Two Back Test |  |
| MEFS/EFgo | Cognitive flexibility/Inhibition/Working Memory | n/a | [info@reflectionsciences.com](mailto:info@reflectionsciences.com) |
| NIH Toolbox | Immediate recall | Auditory Verbal Learning Test | [NIHToolbox.org](https://www.healthmeasures.net/explore-measurement-systems/nih-toolbox) |
|  | Cognitive flexibility/ Executive functioning | Dimensional Change Card Sort |  |
|  | Inhibitory control and attention | Flanker |  |
|  | Working Memory | List Sorting |  |
|  | Language | Oral Reading Recognition |  |
|  | Processing Speed | Pattern Comparison |  |
|  | Episodic memory | Picture Sequence |  |
|  | Language | Picture Vocabulary Test |  |
| **Abbreviations:** *AMES- Assessment of Effort, Motivation, and Self-Regulation; APNA- Adaptable Pediatric Neurocognitive Assessment; BENCI- Batería de Evaluación Neuropsicológica Computerizada Infantil (English translation: Computerized Neuropsychological Battery for Children); CANTAB- Cambridge Neuropsychological Test Automated Battery; EF- Executive Functioning;* *DEEP – DEvelopmental Assessment on an E-Platform; ENABLE- Educational Neuroscience App-Based Learning Environment; n/a: not applicable (as test name describes domain measured); MEFS- Minnesota Executive Function Scale; NIH-National Institutes of Health; PLUS EF- Promoting Learning, Understanding Self-Regulation- Executive Functioning; RACER- Rapid Assessment of Cognitive and Emotional Regulation; START – Screening Tools for Autism Risk using Technology* | | | |
